# Neural Plasticity and Neurogenesis in Mental Disorders

**DOI:** 10.1155/2016/3738015

**Published:** 2016-03-28

**Authors:** Graham Cocks, Mauro G. Carta, Oscar Arias-Carrión, Antonio E. Nardi

**Affiliations:** ^1^Department of Neuroscience, The James Black Centre, King's College London, 125 Coldharbour Lane, London SE5 8AF, UK; ^2^Quality of Care and Translational Medicine, Department of Public Health, Clinical and Molecular Medicine, University of Cagliari, 09042 Monserrato, Italy; ^3^Unidad de Trastornos del Movimiento y Sueño, Hospital General Dr. Manuel Gea González, 14080 Ciudad de México, DF, Mexico; ^4^Laboratory of Panic & Respiration, Institute of Psychiatry, Federal University of Rio de Janeiro, 22410-003 Rio de Janeiro, RJ, Brazil

Adult neurogenesis, the continuous generation of newborn neurons in discrete regions of the brain throughout life, is now widely regarded as a fundamental mechanism of neural plasticity. This phenomenon, and in particular the integration of new neurons into the dentate gyrus of the hippocampus, has been associated with the regulation of important but quite subtle and complex aspects of cognition and memory formation.

In addition to its important role in the healthy adult brain, adult neurogenesis has also been of considerable interest to the research community because of a growing body of literature implicating its deregulation in mental disorders. Perhaps the most high-profile example of this is the putative role of reduced neurogenesis in the adult hippocampus in the pathogenesis of major depression.

However, despite the fact that adult neurogenesis is confined to very discrete regions of the brain, and the established role of adult neurogenesis in major depression notwithstanding, it is becoming increasingly apparent that the deregulation of neurogenesis may impact a much wider range of mental disorders. In this special issue on neural plasticity and neurogenesis in mental disorders, we are pleased to present a series of articles that reflect the broad scope of psychiatric and neurological conditions that are potentially impacted by abnormalities in neurogenesis and neuroplasticity.

L. Varela-Nallar et al. (“Andrographolide Stimulates Neurogenesis in the Adult Hippocampus”) report new data in this issue demonstrating the effects of Andrographolide (ANDRO) on adult hippocampal neurogenesis, a compound the authors have previously identified as a GSK3beta inhibitor. The regulation of beta-catenin by GSK3beta, which is modulated by the Wnt signalling pathway, is well established in playing an important role in regulating neural stem cell proliferation. The authors demonstrate that ANDRO increases adult hippocampal neurogenesis in young and aged mice and in a transgenic mouse model of Alzheimer's disease. Impaired neurogenesis has been associated with early pathological changes in Alzheimer's disease and is also reduced in normal aging. Novel small molecules such as ANDRO that can upregulate adult neurogenesis are therefore of potentially important therapeutic interest in reducing cognitive decline.

D. Feldman et al. (“Developmental Dynamics of Rett Syndrome”) review the role of MeCP2 in abnormal developmental neurogenesis and neural plasticity in Rett syndrome across the lifespan. Rett syndrome is a disorder that has until recently been largely characterized as arising from abnormalities in neural plasticity in postnatal development. In their review article in this issue, D. Feldman et al. also provide insight into the more recent identification of earlier pathological events involving developmental neurogenesis. These earlier effects of MeCP2 loss of function on the generation and integration of neurons in the developing brain are also in line with recent research on other closely related neurodevelopmental conditions such as Autistic Spectrum Disorder where a growing body of literature has also begun to identify aberrant developmental neurogenesis to be an important pathological process, in addition to abnormalities in activity-dependent synaptic plasticity postnatally. Such insights will be important for developing therapeutic strategies to target abnormalities in different aspects of neuronal dysfunction in Rett syndrome arising from these different stages of development.

Defects in synaptic plasticity have been implicated in the pathogenesis of Schizophrenia. J. Gonzalez-Heydrich et al. (“N100 Repetition Suppression Indexes Neuroplastic Defects in Clinical High Risk and Psychotic Youth”) present original data to begin to validate auditory N100 adaptation as a biomarker of clinical high risk and progression to psychosis individuals. Developing biomarkers such as this as an indication of abnormal neural plasticity, particularly in the prodromal stage of psychosis, will be of great value in assessing therapeutic strategies in clinical trial settings in the future.

Despite the considerable progress being made in understanding the role of adult neurogenesis and neuroplasticity in mental disorders, in some areas, there is also a great deal of uncertainty about the nature of such associations. These articles therefore reflect upon and elucidate what is currently known but also often highlight the uncertainty that exists and the continuing work that needs to be done to understand these associations. In this respect, G. Perna et al. (“Are Anxiety Disorders Associated with Accelerated Aging? A Focus on Neuroprogression”) present a valuable novel review of the literature looking at the association of anxiety disorders and aging. This review examines a wide range of potential consequences of anxiety disorder from reduced adult neurogenesis and altered neuroplasticity through to increased beta-amyloid production, telomere shortening, oxidative stress, and chronic inflammation. The paper highlights the need for more work to be undertaken to establish these potentially very serious consequences of the already debilitating condition of anxiety disorder.

Finally, A. A. Marques et al. (“Gender Differences in the Neurobiology of Anxiety: Focus on Adult Hippocampal Neurogenesis”) present an insightful review of gender differences in anxiety and potential differences in adult hippocampal neurogenesis between the sexes. This review provides a useful insight into the neurobiological processes that influence these differences and the important implications for the use of animal models of anxiety disorders.

The contributors to this special issue provide a valuable snapshot of the range of mental disorders associated with abnormalities in neural plasticity and neurogenesis. These articles provide important insights into our current understanding of the role of neural plasticity and neurogenesis in mental disorders, highlighting the current gaps in our knowledge and providing a valuable perspective on the future directions of the field.

*Graham Cocks* *Graham Cocks* *Mauro G. Carta* *Mauro G. Carta* *Oscar Arias-Carrión* *Oscar Arias-Carrión* *Antonio E. Nardi* *Antonio E. Nardi*

